# Association of Genetic Variants Influencing Lipid Levels with Coronary Artery Disease in Japanese Individuals

**DOI:** 10.1371/journal.pone.0046385

**Published:** 2012-09-26

**Authors:** Fumihiko Takeuchi, Masato Isono, Tomohiro Katsuya, Mitsuhiro Yokota, Ken Yamamoto, Toru Nabika, Kazuro Shimokawa, Eitaro Nakashima, Takao Sugiyama, Hiromi Rakugi, Shuhei Yamaguchi, Toshio Ogihara, Yukio Yamori, Norihiro Kato

**Affiliations:** 1 Department of Gene Diagnostics and Therapeutics, Research Institute, National Center for Global Health and Medicine, Tokyo, Japan; 2 Department of Clinical Gene Therapy, Osaka University Graduate School of Medicine, Suita, Japan; 3 Department of Geriatric Medicine and Nephrology, Osaka University Graduate School of Medicine, Suita, Japan; 4 Department of Genome Science, Aichi-Gakuin University, School of Dentistry, Nagoya, Japan; 5 Department of Molecular Genetics, Medical Institute of Bioregulation, Kyushu University, Fukuoka, Japan; 6 Department of Functional Pathology, Shimane University School of Medicine, Izumo, Japan; 7 Division of Endocrinology and Diabetes, Department of Internal Medicine, Nagoya University Graduate School of Medicine, Nagoya, Japan; 8 Department of Diabetes and Endocrinology, Chubu Rosai Hospital, Nagoya, Japan; 9 Institute for Adult Diseases, Asahi Life Foundation, Tokyo, Japan; 10 Department of Internal Medicine III, Shimane University School of Medicine, Izumo, Japan; 11 Morinomiya University of Medical Sciences, Osaka, Japan; 12 Mukogawa Women's University Institute for World Health Development, Nishinomiya, Japan; National Cerebral and Cardiovascular Center, Japan

## Abstract

**Background/Objective:**

In Japanese populations, we performed a replication study of genetic loci previously identified in European-descent populations as being associated with lipid levels and risk of coronary artery disease (CAD).

**Methods:**

We genotyped 48 single nucleotide polymorphisms (SNPs) from 22 candidate loci that had previously been identified by genome-wide association (GWA) meta-analyses for low-density lipoprotein cholesterol (LDL-C), high-density lipoprotein cholesterol (HDL-C), and/or triglycerides in Europeans. We selected 22 loci with 2 parallel tracks from 95 reported loci: 16 significant loci (*p*<1×10^−30^ in Europeans) and 6 other loci including those with suggestive evidence of lipid associations in 1292 GWA-scanned Japanese samples. Genotyping was done in 4990 general population samples, and 1347 CAD cases and 1337 controls. For 9 SNPs, we further examined CAD associations in an additional panel of 3052 CAD cases and 6335 controls.

**Principal Findings:**

Significant lipid associations (one-tailed *p*<0.05) were replicated for 18 of 22 loci in Japanese samples, with significant inter-ethnic heterogeneity at 4 loci–*APOB*, *APOE-C1*, *CETP*, and *APOA5*–and allelic heterogeneity. The strongest association was detected at *APOE* rs7412 for LDL-C (*p* = 1.3×10^−41^), *CETP* rs3764261 for HDL-C (*p* = 5.2×10^−24^), and *APOA5* rs662799 for triglycerides (*p* = 5.8×10^−54^). CAD association was replicated and/or verified for 4 loci: *SORT1* rs611917 (*p* = 1.7×10^−8^), *APOA5* rs662799 (*p* = 0.0014), *LDLR* rs1433099 (*p* = 2.1×10^−7^), and *APOE* rs7412 (*p* = 6.1×10^−13^).

**Conclusions:**

Our results confirm that most of the tested lipid loci are associated with lipid traits in the Japanese, further indicating that in genetic susceptibility to lipid levels and CAD, the related metabolic pathways are largely common across the populations, while causal variants at individual loci can be population-specific.

## Introduction

Plasma lipid levels are heritable risk factors for cardiovascular disease [1]. It has been revealed that a number of genes and pathways are involved in the pathogenesis of Mendelian dyslipidemic syndromes and also contribute to inter-individual variation in plasma lipid levels [2]–[5]. Recent genome-wide association (GWA) studies have identified genetic determinants of plasma lipids primarily, with around 100 genetic loci showing reproducible evidence of association with circulating low-density lipoprotein cholesterol (LDL-C), high-density lipoprotein cholesterol (HDL-C), and/or triglycerides (TG) [5]–[16]. An appreciable part of the loci identified by GWA studies are in or near genes previously known to influence plasma lipid levels, whereas others are not; that is, they are located near genes not previously implicated in lipoprotein metabolism or in the intergenic regions. As these GWA studies were conducted almost exclusively in populations of European descent, studies in non-European populations will allow us to assess the relevance of the findings to other ethnic groups [17]–[19]. Some variants may be more common in specific ethnic groups, thereby providing greater statistical power, or the effects of genetic variants on lipid levels may be enlarged in specific ethnic groups, presumably due to substantial differences in lifestyle factors (e.g., diet) [20].

Epidemiological studies have provided evidence for association between circulating levels of plasma lipids and risk of coronary artery disease (CAD) [21]. A causal role in CAD for LDL-C has been equivocally established via clinical trials using the HMG-CoA reductase inhibitor (or statin) [22], whereas that for HDL-C and TG remains uncertain. In this context, several genetic studies have systematically investigated associations between SNPs at lipid loci and risk of CAD [23], [24]; some of them showed robust CAD association, while others with comparable magnitudes of lipid–SNP association showed inconsistent magnitudes of CAD association. It is assumed that such inconsistency is explained by pleiotropic actions of the lipid loci in part [9], [25].

In the present study, to test associations between lipid traits (LDL-C, HDL-C, and TG) and CAD for SNPs from 22 candidate loci recently reported by GWA studies and their meta-analyses in populations of European descent [5]–[16], we performed a replication study in Japanese populations.

## Materials and Methods

### Ethics Statement

All human participants provided written informed consent, and the ethics committees of the National Center for Global Health and Medicine (NCGM), Kyushu University, Osaka University, Aichi-Gakuin University, Shimane University, Nagoya University, Chubu-Rosai Hospital, Institute for Adult Diseases, Asahi Life Foundation, and Amagasaki Health Medical Foundation approved the protocols.

### Study Populations

#### Lipid Association Study

A replication study of the previously identified variants at 22 candidate lipid loci was performed in the general Japanese population; from 5745 individuals consecutively enrolled in the population-based setting, which is known as Amagasaki Study [26], 4990 individuals without receiving current treatment for dyslipidemia were included in the present study (Table 1). Here, as an approach to *in silico* selection of target SNP loci for the replication study, we used the lipid association results for 1292 Japanese GWA-scanned samples (see Note S1).

**Table 1:** Baseline characteristics of participants in the present study.

	Amagasaki Study panel	GWA-scanned panel	CAD case-control study panel			
			Tier 1		Tier 2	
			Cases	Controls	Cases	Controls
*n*	4990	1292	1347	1337	3052	6335
% of female	39.9	39.2	22.4	44.7	22.3	41.1
Body mass index (kg/m^2^)	22.9±3.2	23.4±3.2	23.8±3.2	23.4±3.2	23.7±3.0	23.0±2.9
Mean age at recruitment (y)	49.0±12.3	65.8±8.0	66.3	65.6	62.7	62.4
Mean age at first event (y)	–	–	63.3	–	62.7	–
Former or current smoker (%)	44.0	45.7	65.5	41.3	65.7	44.1
Hypertension (%)	21.8	45.3	65.2	44.8	53.7	50.1
Diabetes mellitus (%)	5.9	28.4	47.9	25.5	37.9	16.7
Dyslipidemia (%)	42.2	46.1	56.7	54.8	51.9	49.9
LDL-C, mg/dL	123.8±31.4	122.9±30.9	107.1±29.7	124.1±30.9	–	–
HDL-C, mg/dL	62.8±17.7	59.6±16.9	51.2±14.1	61.0±16.6	–	–
Triglycerides, mg/dL	110.5±86.5	118.3±64.2	155.1±83.9	118.7±68.9	–	–
Alcohol drinking			–	–	–	–
None or abstainer (%)	24.6	53.6	–	–	–	–
Drinker (%)	75.3	46.4	–	–	–	–

Plus–minus values are means ± SD.

Diabetes, hypertension, and dysipidemia were identified as risk factors on the basis of the meeting of diagnostic criteria or the receipt of treatment for these conditions (Note S1).

In the GWA-scanned panel, 414 individuals were from the Amagasaki Study panel; only the latter panel was included and analyzed in the current replication study.

#### Case-Control Study for CAD

Case-control study for CAD was performed in a 2-tier design. Detailed characteristics of the individuals analyzed in each tier are described in Table 1 and Note S1. All participants are of Japanese ancestry. Cases were enrolled from clinical practices or annual medical checkups at medical institutions and university hospitals in accordance with the uniformly defined criteria [27]. These criteria included (i) a validated history of either myocardial infarction (MI) or coronary revascularization (coronary artery bypass grafting or percutaneous coronary intervention), or (ii) subjective symptoms of angina pectoris with 1 or more major coronary vessels showing ≥75% stenosis documented by coronary angiography. Controls were individuals randomly selected from a cross-sectional study of cardiovascular risk factors in the recruitment areas and were deemed free of MI by history, physical examination, and electrocardiogram.

Fasting blood samples were collected after ≥6 hours fast for lipid association study. LDL-C was calculated using the Friedewald formula, with missing values assigned to individuals with TG >400 mg/dl.

### SNP Selection and Genotyping

A total of 22 loci were selected with 2 parallel tracks from 95 significant loci that had been identified for Europeans (Table S1) [5]–[16]. First, we selected 16 (of 22) loci showing strong association (*p*<1×10^−30^) in a large-scale GWA meta-analysis [5]; for these 16 loci, we made a detailed search of index SNP markers principally through the lipid GWA studies published to date. Second, we selected 6 other loci that showed reproducible evidence of lipid and CAD associations in Europeans [28]–[30] or suggestive evidence of lipid associations (one-tailed *p*<0.05) in a preliminary screening among 1292 GWA-scanned Japanese samples, which also allowed us to pick up additional index SNP markers at the candidate loci (Note S1 and Table S1).

In this preliminary screening of target SNP loci, genotyping was performed with the Infinium HumanHap550 or HumanHap610-Quad BeadArray (Illumina, San Diego, CA, USA). Data cleaning and analysis were performed using PLINK [31] as described elsewhere [32]. The lambda value for the genomic control in the lipid GWA scan was 1.00 to 1.02, indicating the absence of systematic confounding, such as population stratification, in the GWA-scanned samples (Table 1 and Note S1).

In the main replication study, a total of 48 SNPs were genotyped using the TaqMan assay (Life Technologies Japan, Tokyo, Japan) in the Amagasaki Study panel and the tier-1 CAD case-control study panel (Table S2). Here, part of the tier-1 CAD case-control study samples were characterized in our previous GWA scan for CAD [27] and the genotype data were used for the present study when available.

Further, 9 SNPs were genotyped in the tier-2 CAD case-control study panel to follow up CAD associations in the tier-1 CAD case-control study panel; (1) because they had been robustly confirmed in GWA studies of CAD in European-descent populations or (2) because they showed a concordant tendency of association with both lipid levels and CAD (an arbitrary threshold of odds ratio [OR] >1.2 set in the tier-1 CAD case-control study) in the Japanese.

The genotypic distribution of all tested SNPs was in Hardy–Weinberg equilibrium (*p*>10^−3^). We obtained successful genotyping call rates of >99% for the whole characterized sample.

### Statistical Analyses

#### SNP Association Analysis

The SNPs were tested for association with lipid levels and CAD by using linear regression analysis in an additive genotype model and the Cochran-Armitage trend test, respectively. In the linear regression models, we adjusted continuous lipid traits for age, age^2^, sex, diabetes status and the sample enrollment site (GWA-scanned panel), or for age classes separately by sex, and body mass index (BMI) (Amagasaki study). Age classes were defined according to age distribution in the panel, and included ≤40, 41–50, 51–60 and >60 years. HDL-C and TG were log-transformed before linear regression analysis. In our replication study, one-tailed *p*<0.05 (i.e., two-tailed *p*<0.1) was considered statistically significant for the loci previously shown to have genome-wide significant (*p*<5×10^−8^) association in Europeans [5]–[16]; for an association to be considered significant, it had to involve the same risk allele as that reported in Europeans and was accordingly assessed with the one-tailed test. Otherwise, a significance level was set at *p*<0.05 after adjustment for multiple testing with Bonferroni's correction. For the purpose of uniformity, two-tailed *p* values are shown throughout the text, unless otherwise indicated. For the CAD case-control study, cases and controls were pooled from multi-tier panels (Note S1). We used PLINK [31], the R software (version 2.10.0; http://cran.r-project.org/), and the rmeta and meta packages to test for the associations.

#### Test of Ethnic Diversity

The per-allele effect size, β, of an SNP on lipid levels was compared between the ethnic groups. Lipid levels were standardized as a *z*-score within each ethnic group before cross-population comparison. The interaction of effect estimates with ethnicity (Japanese *vs.* European) was analyzed by Cochran's Q-test (Table S3) [33].

#### Stepwise Regression Analysis for Testing of Independent Associations

To test the most likely explanation for the signals of association among the index SNPs and their genotyped correlates, we performed stepwise linear regression analysis for lipid levels by forward selection (Table S4). If two or more SNPs simultaneously included in the model each attained significance (*p*<0.05), they could have independent associations. To collectively assess the proportion of variance for lipid levels, explained by an SNP, we calculated the coefficient of determination (*R* ^2^) as previously described [16].

## Results

### Preliminary GWA Scan for Lipid Traits

We found no genome-wide significant association in the Japanese GWA-scanned panel (Table 1, Note S1 and Figure S1). Using this screening data set, we then examined association signals at 95 unique loci for which significant evidence of association was previously identified in Europeans [5]–[16]. A total of 8 SNPs at 5 unique loci–*AFF1*, *LPL*, *ABCA1*, *BUD13–APOA1–APOA5*, and *SCARB1*–showed a tendency of association in the GWA-scanned panel, within 50 kb of index SNP markers previously reported for individual loci. They were subjected to the main replication study, as well as 40 candidate SNPs that were chosen from the published studies (Table S1).

### Replication of Selected SNPs in the Japanese

In the current replication study, we tested associations of 48 SNPs from 22 unique loci with LDL-C, HDL-C, and TG in the Amagasaki Study panel (Table S2). The strongest association signals were detected at *APOE-C1* rs7412 for LDL-C, *CETP* rs3764261 for HDL-C, and *BUD13-APOA1-A5* rs662799 for TG, all of which attained a genome-wide significance level (*p*<5×10^−8^). For all the 16 (of 22) loci robustly confirmed in Europeans, genetic association with a lead lipid trait was successfully replicated for index SNP markers (*p* = 5.8×10^−54^–0.042). For the remaining 6 loci, LDL-C association was replicated at *ABO* rs507666 (*p* = 1.2×10^−5^) and TG association was replicated at *AFF1* rs442177 (*P* = 0.045); at *PCSK9*, *KLF14*, *HNF1A*, and *SCARB1*, the direction of association was concordant with that of previous reports [5], although the associations did not attain statistical significance. Further, at 8 (of 22) tested loci–*APOB*, *LPL*, *ABCA1*, *BUD13-APOA1-A5*, *LIPC*, *CETP*, *LDLR*, and *APOE-C1*, we examined lipid-trait association of 3–6 SNPs per locus that were not in close linkage disequilibrium (LD) with each other (in principle, LD coefficient *r^2^*<0.4) and found that multiple SNPs showed independent association with an identical lipid trait at individual loci (Table S4).

**Figure 1:**
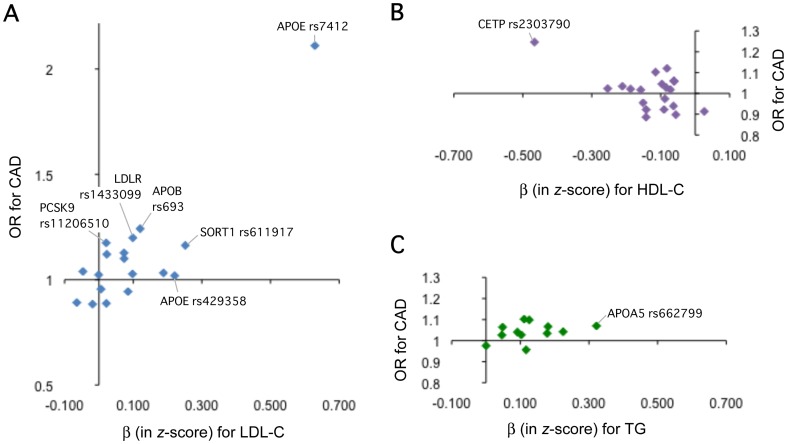
Correlation of effect sizes for CAD risk and 3 lipid traits–LDL-C (a), HDL-C (b), and TG (c)–at SNPs tested for replication in the current study. Genetic impacts on lipid level (β in *x*-axis) and CAD risk (OR in *y*-axis) are compared for the SNPs that were previously reported to associate with the corresponding (lead) lipid trait in Europeans: 18 SNPs for LDL-C (a), 20 SNPs for HDL-C (b), and 12 SNPs for TG (c), where 3 SNPs at *LPL* are included in both (b) and (c). The names of SNPs that were genotyped in the tier-2 CAD case-control study panel are denoted in the plots. For the purpose of readability, error bars are not shown at the individual SNP loci in the figure. See details about the individual SNP loci in Table S2.

Subsequently, we tested CAD associations of 48 SNPs in the tier-1 CAD case-control study panel (Table S2). Two SNPs–*LDLR* rs1433099 (*p* = 0.003) and *APOE* rs7412 (*p* = 7.1×10^−8^)–showed some evidence (an arbitrary threshold of *p*<0.05) of association with CAD in the directions concordant with those previously reported [34], [35]. To evaluate the correlation of effect sizes between lipid traits and CAD, we then depicted scatter plots (Figure 1) and found that 2 other SNPs (*APOB* rs693 and *CETP* rs2303790) showed effect sizes larger than that of *LDLR* rs1433099 (OR≥1.20). We followed up a total of 9 SNPs including these 4 SNPs in the tier-2 CAD case-control study panel (Table 2). In the combined samples (4399 cases and 7672 controls), significant (*p*<0.0056≈0.05/9 SNPs) association was detected for 4 SNPs: *SORT1* rs611917 (*p* = 1.7×10^−8^, OR = 1.37), *APOA5* rs662799 (*p* = 0.001, OR = 1.09), *LDLR* rs1433099 (*p* = 2.1×10^−7^, OR = 1.17) and *APOE* rs7412 (*p* = 6.1×10^−13^, OR = 1.69). At *APOE*, the strength of CAD association was also highly significant in isoform-based comparison (E3/E3 *vs.* E2 carriers; *p* = 3.2×10^−13^, OR = 1.80).

**Table 2:** Coronary artery disease susceptibility loci extensively tested in the present Japanese study.

		Position (Build 36)	Nearby gene(s)	Alleles (coded/other) c	Coded allele freq. in JPN d	Tier 1			Tier 2			Combined				
SNP	Chr					OR	*P*	*N*	OR	*P*	*N*	Coded allele freq.		OR	*P*	*N* total
						(95% CI)			(95% CI)			Case	Control	(95% CI)		
rs11206510	1	55,268,627	*PCSK9*	T/C	0.95	1.18	0.193	2,683	0.98	0.768	9,366	0.952	0.952	1.01	0.833	12,049
						(0.92–1.51)			(0.85–1.13)					(0.89–1.15)		
rs611917	1	109,616,775	*SORT1*	A/G	0.92	1.16	0.172	2,592	1.45	1.6E-08	9,376	0.942	0.922	1.37	1.7E-08	11,968
						(0.93–1.45)			(1.27–1.65)					(1.23–1.53)		
rs693	2	21,085,700	*APOB*	A/G	0.05	1.24	0.112	2,682	1.05	0.496	9,374	0.048	0.044	1.08	0.219	12,056
						(0.95–1.63)			(0.91–1.22)					(0.95–1.23)		
rs662799	11	116,168,917	*APOA5*	G/A	0.29	1.07	0.233	2,683	1.11	1.9E-03	9,376	0.352	0.332	1.09	0.001428404	12,059
						(0.95–1.20)			(1.04–1.18)					(1.03–1.16)		
rs2259816 a	12	119,919,970	*HNF1A*	T/G	0.64	0.87	0.016	2,676	0.95	0.099	9,375	0.536	0.553	0.94	0.015	12,051
						(0.78–0.98)			(0.89–1.01)					(0.89–0.99)		
rs2303790	16	55,574,793	*CETP*	A/G	0.96	1.25	0.136	2,662	1.22	0.033	9,375	0.972	0.966	1.20	0.020	12,037
						(0.92–1.70)			(1.01–1.47)					(1.03–1.40)		
rs1433099	19	11,103,658	*LDLR*	C/T	0.70	1.20	2.9E-03	2,684	1.16	1.4E-05	9,382	0.739	0.708	1.17	2.1E-07	12,066
						(1.06–1.35)			(1.09–1.25)					(1.10–1.24)		
rs7412	19	50,103,919	*APOE*	C/T	0.95	2.11	7.1E-08	2,658	1.60	4.7E-08	9,378	0.970	0.951	1.69	6.1E-13	12,036
						(1.59–2.82)			(1.35–1.90)					(1.46–1.95)		
rs429358	19	50,103,781	*APOE*	C/T	0.10	1.02	0.809	2,650	0.94	0.263	9,382	0.103	0.108	0.95	0.240	12,032
						(0.85–1.23)			(0.85–1.04)					(0.87–1.04)		
E3/E3 vs. E2	19		*APOE*			2.31	4.1E-08	2,157	1.65	6.0E-08	7,458	0.949	0.916	1.80	3.2E-13	9,615
carriers b						(1.69–3.23)			(1.37–2.00)					(1.54–2.13)		

A cororary artery disease (CAD) association study comprises two-tiered sample; the tier-1 was done in 1347 cases and 1.337 controls and the tier-2 done in 3,052 cases and 6.335 controls. Association results from the two tiers were combined by pooling the genotype counts.

a rs735396 was genotyped in replacement for rs2259816 in the GWA study panel (*r2* = 1.000 to rs735396 in HapMap JPT+CHB). Rs735396 is in LD with rs1169300 (*r2* = 0.783 in HapMap JPT+CHB), which was tested for LDL-C association. The direction of association with CAD risk appears to be opposite to that for increased LDL-C in the *HNF1A* locus.

b Following the previous meta-analysis of CAD association with ApoE genotype (Benett et al. JAMA 2007, ref. 35), CAD risk was compared between E3/E3 individuals and E2 carriers (excluding E2/E4).

c Alleles are nominated as those in dbSNP Build 130 mapped on the strand of Human Genome Build 36.3.

d Allele frequencies in the Japanese general population from GeMDBJ (*n* = 964) or HapMap JPT (*n* = 90; rs662799, rs2259816, rs2303790, and rs7412) or Amagasaki Study panel (rs429358).

### Ethnic Heterogeneity in Effect Sizes for Lipid Traits

In the current cross-population comparison, lipid trait association data were available for 43 of 48 tested SNPs (Table S3). Of 43 SNPs, the direction of association was concordant at 40 loci (93%) between 2 ethnic groups. All of 3 inverted SNPs–*APOB* rs515135, *APOB* rs754523, and *APOE-C1* rs4803750–were previously reported to associate with LDL-C in Europeans and were found to show significant inter-ethnic heterogeneity (*p* = 3.9×10^−9^–2.8×10^−4^, Table S3). While the direction of association was concordant between the ethnic groups, 4 other SNPs–*APOE* rs7412 (for LDL-C), *CETP* rs1800775 (for HDL-C), *APOB* rs676210 and *BUD13-APOA1-A5* rs662799 (both for TG)–also showed significant inter-ethnic heterogeneity (*p* = 7.5×10^−5^–0.0016) after adjustment for multiple testing (*p*<0.0023≈0.05/22 loci). Despite a relatively high correlation coefficient (*r* = 0.723) for 48 SNPs as a whole, effect allele frequencies at several SNPs substantially differed between the ethnic groups.

**Figure 2:**
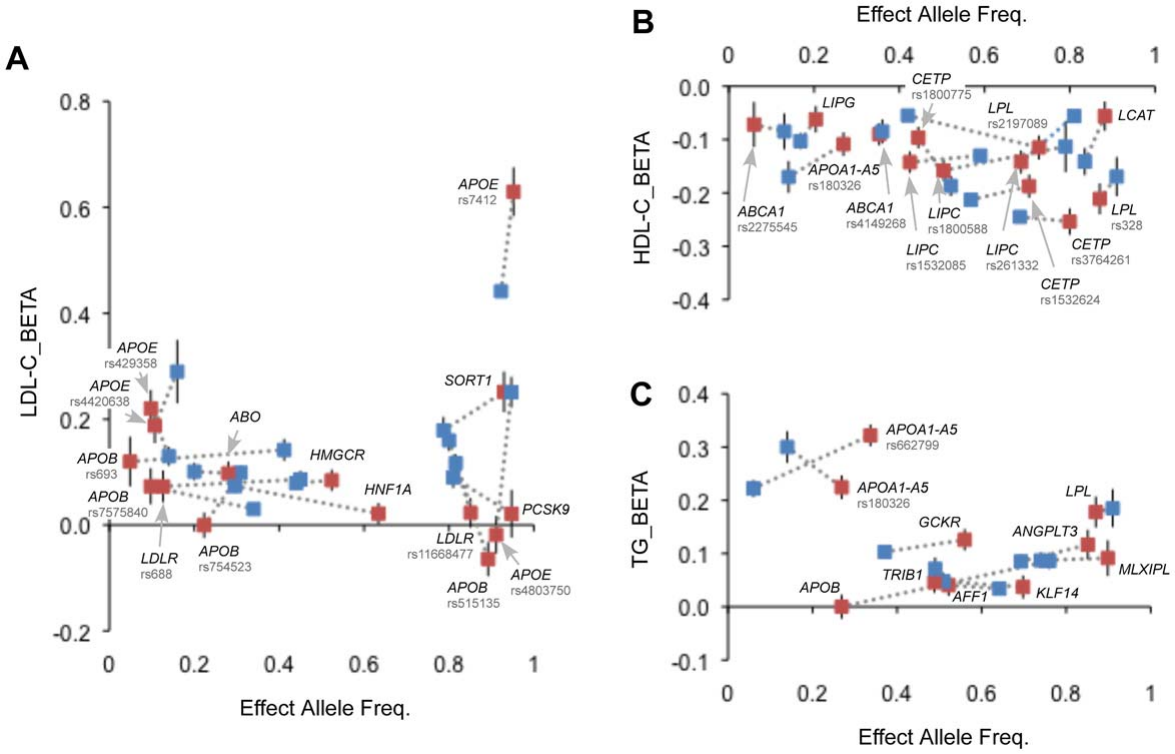
Cross-population comparison of per-allele effect of SNPs associated with LDL-C (a), HDL-C (b), and TG (c) between the Japanese and European-descent populations. Effect alleles are defined as those that increase LDL-C or TG or that decrease HDL-C. The effects of each variant on lipid traits are shown by squares, colored in red (Japanese) and blue (Europeans). The gray dotted lines between the red and blue squares represent an identical locus. See details about the individual SNP loci in Table S3.

We further assessed the strength of association for individual SNPs by measuring *R* ^2^, which is scaled against effect size and effect allele frequency in Figure 2. We found that the same level of statistical significance is detectable at some loci in one population with a much smaller sample size than that in the other population, i.e., the presence of inter-ethnic heterogeneity in *R* ^2^ (defined by both effect size and effect allele frequency). For example, the strength of association with LDL-C was more prominent in Europeans at *PCSK9* rs11206510, *APOB* rs515135, *APOB* rs754523, *LDLR* rs11668477, and *APOE-C1* rs4803750 than in the Japanese (Figure 2).

### Evaluation of Allelic Heterogeneity for Lipid Traits

At 5 loci, we verified statistical independence of multiple lipid-associated variants by regression analysis (Table S4; see Methods). In this process, we identified a novel genome-wide significant SNP, rs3741301 (*p* = 2.7×10^−14^), which is one of the variants independently associated with TG at *BUD13-APOA1-A5*. We also verified two genome-wide significant SNPs, *LIPC* rs11858164 (*p* = 4.0×10^−10^) and *CETP* rs2303790 (*p* = 1.1×10^−16^), both of which were previously reported to show suggestive evidence of association with HDL-C [36], [37].

We estimated cumulative effects of genetic variants on lipid traits by considering the presence of multiple independently-associating SNPs at individual loci. Percentages of the variance (*R* ^2^) jointly explained by the tested SNPs were 7.8% for LDL-C, 10.7% for HDL-C, and 8.8% for TG in the current Japanese study.

## Discussion

The present study systematically investigated genetic susceptibility to lipid levels and its relevance to CAD in Japanese populations. From the viewpoint of population genetics, generalization of lipid association results previously identified in European GWA studies (or meta-analyses) to non-European populations is an issue of interest, because it can facilitate the fine mapping of common causal variants by providing clues to whether SNPs identified in European GWA studies are simply tag-SNP or “synthetic association” markers [38], [39] or are more likely to be true functional variants. Thus far, only a few studies have addressed this in non-European populations [17], [18], [40], and our study is the first replication study in east Asians, with focus on both lipid trait and CAD associations.

**Figure 3:**
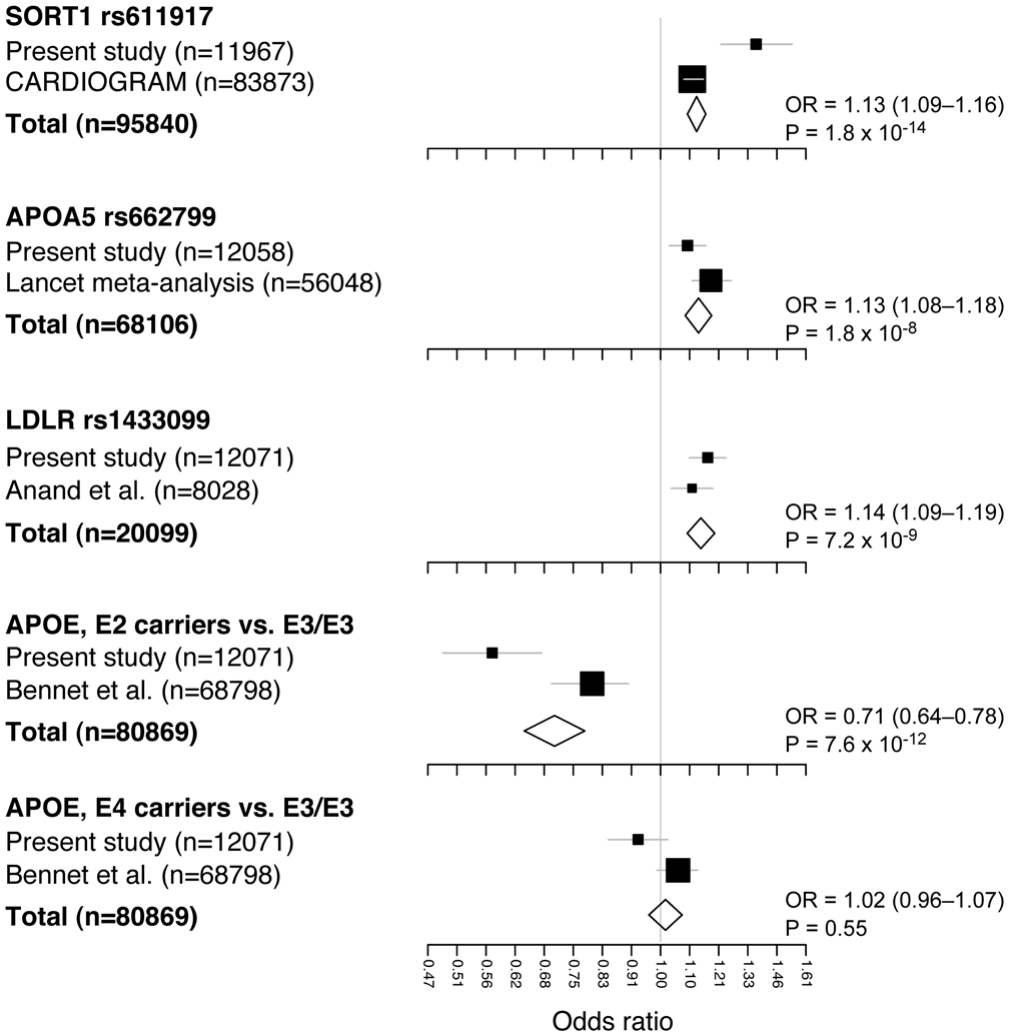
Meta-analysis of CAD association with selected SNPs or variants, including the current and previously reported studies. Effect sizes of *SORT1* and *APOE* variants were heterogeneous between the current study and those previously reported [29], [35]: *p* =  6.8×10^−4^ for *SORT1*, *p* = 1.7×10^−3^ for *APOE* (E2 carriers vs. E3/E3) and *p* = 0.041 for *APOE* (E4 carriers *vs.* E3/E3) by Woolf's test.

Replicating a study of candidate loci previously identified by GWA meta-analyses of European-descent populations [5]–[16], we have found some degree of ethnic diversity in lipid variants, while 18 of 22 tested loci are associated with lipid traits in the Japanese. The loci showing strong lipid associations (e.g., *SORT1* and *APOE-C1* for LDL-C; *LPL*, *LIPC*, and *CETP* for HDL-C; and *GCKR*, *LPL*, and *BUD13-APOA1-A5* for TG) were in good agreement between the ethnic groups except *APOB*, where associations with LDL-C and TG were relatively weak in the Japanese (Table S2). Also, in the present study, we confirmed significant genetic impacts of 4 loci–*SORT1*, *APOA5*, *LDLR*, and *APOE*–on CAD in the Japanese. Of note, the effect size of *APOE* variants on CAD was significantly large in the Japanese (Figure 3). Moreover, it is of interest that as compared with the results for Europeans, the variance for LDL-C levels explained by individual SNP loci tended to be smaller in the Japanese (Figure 2), despite an overall cross-population consistency of genetic variants.

For the most significant locus, *APOE*, 3 major isoforms are known to exist; E2 and E4 isoforms can be differentiated from E3 by rs7412 and rs429358, respectively. In agreement with a previous study of meta-analysis [35], E2 (T-allele of rs7412) and E4 (C-allele of rs429358) exerted decreasing and increasing genetic effects on LDL-C, respectively, as compared to E3. While E2 carriers had a significantly decreased risk of CAD (OR = 0.56; 95% CI, 0.47–0.65; *p* = 3.2×10^−13^), E4 carriers showed no increased risk of CAD (OR = 0.94; 95% CI, 0.85–1.03; *p* = 0.18) in the Japanese population (Figure 3), which is inconsistent with the previous reports [35]. In addition, we found that effect sizes of *APOE* variants were heterogeneous (*p*<0.05) between the current study and those previously reported [11], [35]. The genetic impacts on CAD seem to be more prominent for *APOE* rs7412 than the loci that we previously detected in the Japanese GWA scan (e.g., *CDKN2A/B* on 9p21 and *BRAP/ALDH2* on 12q24) [27]. Because rs7412 and rs429358 themselves and their proxies are not included in the list of SNPs that are assayed by most of the GWA scan platforms, it is likely that these SNPs have failed to be tested for CAD associations in the previous GWA studies [5], [6], [29].

As an approach to examining clinical relevance of lipid-associated SNPs, we assessed whether they are also associated with CAD in a manner consistent with established epidemiological relationships; i.e., SNP alleles that increase LDL-C or TG or that decrease HDL-C should be associated with increased risk of CAD, in proportion to the genetic effects on lipid traits. We inspected correlations between genetic effects on CAD risk and those on lipid traits (Figure 1). When we focus on association between a target SNP and its lead lipid trait, there appears to be a fair correlation between genetic effects on CAD and those on LDL-C (*r* = 0.835). As mentioned above, *APOE* rs7412, located in the far upper-right part of the distribution, showed the strongest association not only with LDL-C but also with CAD (OR = 1.69; 95% CI, 1.46–1.95; *p* = 6.1×10^−13^, in the combined sample). *CETP* rs2303790, located in the far upper-left part of the distribution, also showed the strongest association with HDL-C and a tendency of association with CAD (OR = 1.20; 95% CI, 1.03–1.40; *p* = 0.02) although not significant after adjustment for multiple testing; *APOA5* rs662799, located in the upper-right part of the distribution, showed the strongest association with TG and significant association with CAD (OR = 1.09; 95% CI, 1.03–1.16; *p* = 0.0014). Thus, while it appears to be generally pronounced for LDL-C associated SNPs, the present study has confirmed in the Japanese that SNPs showing association with LDL-C and TG are significantly associated with CAD, as previously reported in Europeans [28]–[30]. Given a suggestive association of *CETP* rs2303790 with CAD, on the other hand, further investigation is warranted to clarify the relevance of HDL-C associated SNPs to CAD risk.

The presence of allelic and ethnic heterogeneity has been rigorously examined for several lipid-associated loci [14], [16]–[18]. For example, in the *APOB* gene, which is one of the most significant LDL-C associated loci in Europeans, Benn [41] has shown that a minimal set of tag-SNPs capturing the entire variation in *APOB* cannot be identified because of the complex LD structure and thus most SNPs must be evaluated separately in association studies and that the magnitude of effect sizes of common, associated SNPs in *APOB* are modest. The present study has confirmed such findings, i.e., the presence of allelic heterogeneity and modest effect sizes, and has further revealed the presence of ethnic heterogeneity in *APOB*. In the Japanese, we have found that the strength of lipid association of SNPs in *APOB* is modest, because of inter-ethnic differences in effect sizes (for rs676210, rs515135, and rs754523) and/or in effect allele frequencies (for rs676210 and rs693) (Tables S3 and S4). As a consequence, an explained variance of an SNP (measured by *R^2^*) that represents the ability to detect association signals in *APOB* is generally smaller in the Japanese than in Europeans (Figure 2). Similarly, the presence of allelic heterogeneity is indicated for 7 other lipid loci (Table S4), among which we have identified 2 new lipid-associated SNPs in the present study, *ABCA1* rs4149263 and *BUD13-APOA1-A5* rs3741301; they show association signals independent of the lead SNPs previously reported at each locus [6]–[9], [36]. Further, with regard to allelic and ethnic heterogeneity, in the Japanese the strongest CAD association at *LDLR* was detected for rs1433099, whereas in Europeans a prominent association was reported for rs6511720 (which is not polymorphic in the Japanese) [5], [12].

Through a series of studies, we demonstrate in the Japanese that 18 loci are associated with lipid traits, in part, in a race/ethnicity-specific manner with some loci being also associated with CAD risk. Physiological candidate genes, known to be involved in lipid metabolism, are located in the vicinity of 15 (of 18) loci (Table S5). A substantial part of the tested loci show significant associations with >1 lipid traits in Europeans and Japanese concordantly, whereas the type(s) of lipid traits may not be consistent with the results obtained in mice lacking the corresponding gene. This brings up some caveats when we interpret the findings in rodent models. Moreover, in a few instances (e.g., *APOA5* and *LDLR*), CAD has been reported as a clinical symptom among the patients with a Mendelian type of lipid disorder (Table S5), which corroborates the biological impacts, even though individually modest, exerted by naturally occurring common variants in humans. As new lipid-associated genes and variants continue to be identified, knowledge about the genetic basis will continue to improve and help us to develop a broader understanding of lipoprotein metabolism and atherosclerosis towards better clinical utility of individual genetic make-up.

## Supporting Information

Note S1 (PDF)

Table S1 A list of SNPs at the lipid-associated loci, previously reported in European GWAS
(XLS)

Table S2 SNPs tested for lipid association in the Japanese
(XLS)

Table S3 Comparison of effect sizes for lipid between Japanese and Europeans
(XLS)

Table S4 Independence of association signals by stepwise regression analysis
(XLS)

Table S5 Lipid and CAD phenotypes of candidate genes, reported in humans and mice
(XLS)

Figure S1 Manhattan plots of GWA scan for LDL, HDL and TG in the Japanese. SNPs are plotted with their physical position and –log_10_(P) in the horizontal and vertical axes, respectively. WGAViewer (Ge *et al.*) was used for the visualization. Suggestive association signals were detected in a preliminary GWA scan: for example, lipid trait associations with *P*<2×10^−6^ were detected for 7 loci including TG association at rs3741301 (*BUD13-APOA1-A5*) on chromosome 11. However, none but rs3741301 have replicated association in the Amagasaki panel.
(TIFF)
