# Supplementary material for: Association of Genetic Variants Influencing Lipid Levels with Coronary Artery Disease in Japanese Individuals
Source: PLoS One. 2012 Sep 26;7(9):e46385. doi: 10.1371/journal.pone.0046385 (PMC3458872; doi:10.1371/journal.pone.0046385)
Supplement: Note S1 — (PDF) [file pone.0046385.s001.pdf]

SUPPLEMENTARY NOTE

Subjects 2

1) Study samples for lipid traits 2

1-1. ***Japanese general population cohort sample: Amagasaki Study*** 2

1-2. ***Genome-wide association (GWA) scanned sample*** 2

2) Study samples for CAD case-control studies 3

SNP typing and quality control 4

1) SNP genotyping 4

2) Quality control of GWA scan data 4

Subjects

1) Study samples for lipid traits

1-1. Japanese general population cohort sample: Amagasaki Study

To investigate lifestyle factors and genetic susceptibility to cardiovascular disease and risk factor traits, we enrolled individuals who sought medical assessment from September 2002 to August 2003 at the Amagasaki Health Medical Foundation, as described elsewhere [26]. The participants were included if they were over 18 years of age and had full clinical examination data, along with a completed questionnaire on their lifestyle. On the basis of these inclusion criteria, 5745 individuals (3435 men and 2310 women) were enrolled in this prospective cohort study, known as the Amagasaki Study. All participants provided written informed consent. Fasting blood samples were collected after ≥6 h fast during a visit to measure glucose and lipid concentrations using standard techniques. People who were receiving current treatment for dyslipidemia and those who did not meet the fasting condition (755 subjects in total) were excluded from the analysis (Table 1).

1-2. Genome-wide association (GWA) scanned sample

Our genetic studies of lipid traits were originally organized as part of the ongoing GWA study for cardiometabolic disorders among the Japanese. After excluding individuals with current treatment for dyslipidemia, 1292 Japanese samples were used for a preliminary screening of lipid trait association. These subjects were enrolled at four separate sites—the Tokyo, Nagoya, Osaka, and Shimane districts. Subjects in the Tokyo district (n=275) were selected from participants in the Hospital-based Cohort Study at the National Center for Global Health and Medicine (NCGM) and outpatients at the Institute for Adult Diseases, Asahi Life Foundation. Subjects in the Nagoya district (n=40) were selected from patients who either visited outpatient clinics of or were admitted to the Nagoya University Hospital and its affiliated medical institutions.

Subjects in the Osaka district (*n*=548) are part of the Japanese general population cohort samples described above and those enrolled from the clinical practice or the annual health checkup of medical institutions. Subjects in the Shimane district (*n*=429) are people who visited the Shimane Institute of Health Science for a health screening examination between July 2003 and March 2007. In all populations, we included the participants' data in the test of lipid trait association, when their blood samples were collected at least after a 6-h fast.

2) Study samples for CAD case-control studies

A total of 2684 Japanese subjects (1347 cases and 1337 controls in the tier-1 panel) were used for testing CAD association of 48 SNPs. The details about the subjects were described previously [27]. The tier-2 panel comprised 9387 unrelated Japanese individuals (3052 cases and 6335 controls) who either visited outpatient clinics of or were admitted to one of the collaborating hospitals, or participated in a health checkup of residents and workers in the Nagoya district. Here, the 3052 CAD patients (2371 men and 681 women) all underwent coronary angiography and left ventriculography. Nine SNPs were genotyped in the tier-2 panel (see the Methods section).

Subjects with systolic blood pressure ≥140 mm Hg, diastolic blood pressure ≥90 mm Hg and/or the current use of antihypertensive medication were categorized as having hypertension. Dyslipidemia was defined according to the Japan Atherosclerosis Society Guidelines: LDL cholesterol ≥140 mg/dl, HDL cholesterol <40 mg/dl, triglycerides ≥150 mg/dl, or undergoing treatment with lipid-lowering drugs. Type 2 diabetes was defined as fasting plasma glucose levels ≥126 mg/dl, HbA1c ≥6.5%, and/or current treatment for diabetes.

For assessing the overall strength of CAD association in the Japanese, we pooled the genotype counts to combine the genetic effects estimated across the multi-tier scan.

SNP typing and quality control

1) SNP genotyping

In the Amagasaki Study panel and CAD case-control study panels, genotyping was performed with the TaqMan assay (Applied Biosystems) unless otherwise indicated. In the GWA study panel, genotyping was performed with Infinium HumanHap550/ Human610-Quad BeadArray (Illumina, San Diego, CA, USA), which interrogated 550K/610K SNPs, according to the manufacturer's protocol. This set of SNP markers reportedly captured 87% of common SNPs with an LD coefficient of $r^2>0.8$ in the HapMap JPT and CHB populations (according to the manufacturer's brochure). Assay accuracy and reproducibility were measured using DNA from CEU samples genotyped as part of the HapMap project [http://www.hapmap.org]. Genotype calling was performed using BeadStudio software (Illumina) and genotype calls with a 'GenCall' Score <0.53 were dropped from the analysis. The GenCall Score measures the reliability of genotype calls based on the clustering of dye intensities (www.illumina.com/downloads/ GenCallTechSpotlight.pdf).

2) Quality control of GWA scan data

Quality control of SNPs and samples was performed as previously described [32]. Briefly, data cleaning and analysis were performed using PLINK software [31]. Among the assayed SNPs, we excluded SNPs for which (1) genotype call rate, <0.95; (2) significant ($P<10^{-6}$) deviation from the Hardy-Weinberg equilibrium; or (3) MAF, <0.01. The remaining 456841 SNPs were analyzed in genome scan. The average call rate for the quality-controlled 456841 SNPs was 99.7% in 1292 samples tested for lipid trait association.
